# A Commemorative Issue in Honor of 200th Anniversary of the Birth of Gregor Johann Mendel: The Genius of Genetics

**DOI:** 10.3390/ijms241411718

**Published:** 2023-07-20

**Authors:** Petr Smýkal, Eric J. B. von Wettberg

**Affiliations:** 1 Department of Botany, Palacky University, 771 47 Olomouc, Czech Republic; 2 Department of Plant and Soil Science and Gund Institute for the Environment, University of Vermont, Burlington, VT 05405, USA

## 1. Introduction

In celebration of the bicentennial of the birth of Gregor Johann Mendel, the genius of genetics, this Special Issue presents seven papers. Mendel’s bicentenary provides an excellent opportunity for presenting recent developments and the latest research in the wide fascinating broad range of today’s biology—plants, animals and microbial kingdoms, from evolutionary to modern breeding approaches. This Special Issue reflects this, with papers across a range of taxa and reflecting the spectrum of techniques utilized in contemporary genetics.

Gregor Johann Mendel has left an indelible mark on the history of science. Born on 20 July 1822 in today’s Czech Republic, Mendel is best known for discovering the basic principles of heredity through cross-breeding and careful examination of garden peas plants. His foundational work in genetics and Darwin’s evolutionary theory established the theoretical basis of today’s biology. Many books and reviews have been written about his life and scientific achievements, and Mendel’s principles of heredity are found in every biology textbook. The wider public broadly understands them. Any genetic study is a celebration of Mendel’s work. Research in genetics impacts our everyday lives in medicine, agriculture and conservation. The rules found by Mendel remain foundational to modern biology.

Mendel’s genius originated from using several novel approaches, such as the use of mathematical and statistical analysis, which were not common in his time prior to his utilizing them. This allowed him to formulate general conclusions on hereditary rules based on exact experiments. Following Mendel’s interdisciplinary approach, the biological sciences are increasingly a collaborative domain, with research projects including aspects of physics, mathematics and chemistry. This is fueled by technical advances such as DNA sequencing, allowing for more discoveries but also creating new challenges, such as data storage, analysis, and predictions. This led to the establishment of systems biology as an integrative approach to understanding complex networks that characterize the phenotypes in the cell that draws techniques and approaches from multiple disciplines.

Over the past 200 years, we have progressed from classical genetics, followed by molecular genetics and epigenetics, to the genomics era and are now entering an era of genome editing. However, despite all those advancements, the methods of classical genetics are still used in biology and form the basis of crop breeding and animal husbandry. Genomics has revolutionized the field of genetics because we can now study nearly all genes in the genome and carry out genetic studies on (almost) any species.

The history of genome engineering goes back almost 70 years to the initial discovery of the DNA double helix. The ability to edit genes became a reality with the discovery of restriction enzymes in the 1970s. As time passed, the need for precision in genome editing became more evident. The discovery of zinc finger nucleases in the 1980s addressed this issue, followed by transcription activator-like effector nucleases (TALENs) in 2011. However, scientists were still searching for an easier and faster way to edit genes and discovered a new genome editing method derived from CRISPR-Cas9. This system has long existed in bacteria to help them fight invading viruses. All these advancements in genome editing techniques have opened up new doors for what genome editing can do to address issues in medicine, agriculture and beyond.

Besides being Mendel’s model organism, the pea (*Pisum sativum*) is the fourth most cultivated pulse worldwide, a source of protein in animal feed and human food. Rispail et al. [1] have examined patterns of diversity and population structure in a core collection of 325 accessions to reflect the diversity of *Pisum* using DARTseq genotyping. The collection includes all *Pisum* species and subspecies and will serve for genome-wide association (GWAS) and genomic selection studies in peas for agronomic traits and disease resistance. The study confirmed that domesticated *P. abyssinicum* is distinct from *P. sativum* and arose from an independent domestication event. The next cluster was formed by wild *Pisum fulvum* and *P. elatius* accessions, while domesticated *P. sativum* groups formed four distinct clusters. This result supported the genetic distinctiveness of the subset of South Asian *P. sativum* accessions, Central Asian accessions (Afghanistan, the Hindu Kush and Himalayan mountains) and, finally, accessions from central areas of China.

Dobrovolná et al. [2] have mined the *Pisum* genome for potential G-quadruplex-forming sequences. These have been long considered rare and physiologically unimportant in vitro curiosities, but recent methodological advances have proved their presence and functions in vivo. In peas, these are not located randomly in the nuclear genome but instead associated with regulatory regions, including 5′UTRs, and upstream of the rRNA regions, and were also found in mitochondrial and chloroplast DNA. Interestingly, G-quadruplex-forming sequences were also associated with specific transposable elements such as TIR and LTR and around them, pointing to their role in their spreading in nuclear DNA.

Surkova and Samsonova [3] have looked for shared determinants of flowering time in legumes. Although the vernalization requirement for exposure to low temperatures to trigger flowering has been mainly studied in *Arabidopsis* model and cereals, it is also required in other crops, including some legumes. The molecular basis of vernalization responses seems to be conserved, based on the MADS-box gene FLOWERING LOCUS C (FLC), a flowering repressor. FLC silencing releases the expression of the main flowering inductor FLOWERING LOCUS T (FT), resulting in a floral transition. However, available genomes have shown that the FLC gene is missing in many legume species, including *Medicago truncatula*, *Pisum sativum*, *Vicia faba*, *Lens culinaris* and *Cicer arietinum*, and genistoid legumes such as *Lupinus angustifolius*, *Lupinus luteus* and *Lupinus albus*. However, FT seems to be preserved and functional, as shown for *Medicago truncatula* and narrow-leafed *lupin Lupinus angustifolius*. The authors review flowering genes in selected cool-season legumes showing that cold-induced de-repression of flowering activators proceeds via different mechanisms than the model plant *Arabidopsis.*

Although soybean seeds consist of approximately 40% protein and 20% oil, about 33% of mature, dry seeds are carbohydrates. Hu et al. [4] have examined the genetic basis of seed sugar composition in soybean using a genome-wide association (GWAS) on a population of 323 soybean germplasm accessions. They focused on soluble sugars such as fructose, glucose, stachyose, verbascose and raffinose. Ten candidate genes on six chromosomes were significantly associated with sugar content. According to gene ontology classification, eight genes were involved in the sugar metabolism in soybean and showed similar functions in *Arabidopsis.*

Mutants are often used to study gene function. Mutations are one of the sources of variation important for the evolutionary adaptation of organisms and crops. In addition to spontaneous mutations used early in genetic research, induced mutations are useful for crop breeding. Deng et al. [5] have mutagenized two garden pea varieties susceptible to powdery mildew and Fusarium wilt by irradiation. The progeny were screened for resistance to *Fusarium* wilt and powdery mildew. A 129 bp fragment deletion in the PsMLO1 gene of five mutants that gained resistance to powdery mildew was identified.

Long-lived, vegetatively propagated trees are incredibly challenging for improvement. Prudencio and colleagues [6] reviewed spontaneous mutations used frequently in tree breeding. These are based on errors during DNA replication, damage from reactive oxygen species (ROS) and activity of transposable elements (TE). The mutation rate can be increased by using physical or chemical mutagens, which are used to increase somaclonal variation in the tissue culture of fruit trees. Finally, the authors review the use of newly emerging gene editing tools to look for mutations in *Prunus.* All these have been shown in relation to critical agronomic traits, including winter dormancy and flowering time, flower self-compatibility and fruit quality.

The contribution of Kim et al. [7] is to human genomics. More than 6000 traits and disorders have been documented in humans, inherited according to Mendel’s laws and controlled by a single gene. With the discovery of the genetic basis of disease comes improved diagnosis, the potential for prenatal screening, and the development of new therapies for the condition. However, most traits in biology, including many common human diseases, such as diabetes and inflammatory disorders, show more complex, polygenic inheritance. In addition, many genes and environmental factors contribute to the risk of developing disease. These authors have looked for metabolic disorders, including obesity, abdominal obesity, hypertension, type 2 diabetes, hypercholesterolemia, hypertriglyceridemia, hypo-high-density lipoprotein cholesterolemia and metabolic syndrome in a Korean population using an impressive set of over 50 thousand genome-wide sequenced individuals. Genome-wide association study (GWAS) resulted in the identification of 101 significant loci for eight metabolic phenotypes.

Rounding out the Special Issue, Vescheti et al [8] propose a comprehensive perspective on genomic instability, integrating mutations, chromosomal rearrangements, telomeric shortening, and epigenetic alterations. The review is very timely, as methods to characterize various forms of mutations that are not single nucleotide polymorphisms have rapidly matured in recent years, showing the breadth and frequency of genomic structural variation and epigenomic variation. The authors start with a review of mechanisms to defend against and repair different types of mutations and genomic changes. They then examine a range of complex diseases and phenotypes, such as aging, which are linked to telomeric shifts, and then move to examine our growing understanding of the impacts of variation in epigenomic regulation. The work is a timely tribute to the groundbreaking work of Mendel due to the effort to synthesize our understanding of genomic instability from the scale of nucleotides to whole chromosomes.

## 2. Conclusions

All seven papers illustrate the advancement of genetics since Mendel´s time, particularly thanks to low-cost, high-throughput DNA sequencing. Mendel postulated transmissible factors, genes, to explain the inheritance of traits. He discovered that these factors, genes, exist in different forms, which we now call alleles. We moved from mapping the human genome, an international endeavor that took over a decade and cost billions of dollars, to sequencing individual genomes for a mere fraction of the cost in a relatively short time. This impacted all aspects of today’s biology. But the real promise of genome sequencing lies in true population-scale sequencing, ultimately at the scale of millions of individuals. Human genomics is leading the field, followed by animals and plants. On the other hand, to make sense of DNA sequences, we need to link them to the phenotypic traits, one which Mendel scored. Only then can we have an understanding of genomic information. Over time, evidence of biological complexity has emerged, adding epigenomics and transcriptional, translational regulatory steps. Moreover, additional players, including small RNAs and long non-coding RNAs, violate once-established scenarios of gene regulation to be the exclusive province of proteins.
